# Lesion network mapping of mania using different normative connectomes

**DOI:** 10.1007/s00429-022-02508-8

**Published:** 2022-05-16

**Authors:** Gonçalo Cotovio, Francisco Faro Viana, Michael D. Fox, Albino J. Oliveira-Maia

**Affiliations:** 1 Champalimaud Research and Clinical Centre, Champalimaud Foundation, Lisbon, Portugal; 2 Department of Psychiatry and Mental Health, Centro Hospitalar de Lisboa Ocidental, Lisbon, Portugal; 3 NOVA Medical School, NMS, Universidade Nova de Lisboa, Lisbon, Portugal; 4 Department of Physics, Faculdade de Ciências da Universidade de Lisboa, Lisbon, Portugal; 5 Center for Brain Circuit Therapeutics, Departments of Neurology, Psychiatry, and Radiology, Brigham and Women’s Hospital, Harvard Medical School, Boston, MA USA; 6 Athinoula A. Martinos Centre for Biomedical Imaging, Department of Neurology and Radiology, Massachusetts General Hospital, Charlestown, MA USA; 7 Berenson-Allen Center for Non-Invasive Brain Stimulation and Division of Cognitive Neurology, Department of Neurology, Beth Israel Deaconess Medical Center, Harvard Medical School, Boston, MA USA

**Keywords:** Lesion network mapping, Connectome, Reproducibility, Mania

## Abstract

Lesion network mapping is a neuroimaging technique that explores the network of regions functionally connected to lesions causing a common syndrome. The technique uses resting state functional connectivity from large databases of healthy individuals, i.e., connectomes, and has allowed for important insight into the potential network mechanisms underlying several neuropsychiatric disorders. However, concerns regarding reproducibility have arisen, that may be due to the use of different connectomes, with variable MRI acquisition parameters and preprocessing methods. Here, we tested the impact of using different connectomes on the results of lesion network mapping for mania. We found results were reliable and consistent independent of the connectome used.

## Introduction

Lesion network mapping (LNM) was developed as a technique to address potential network mechanisms underlying a variety of neuropsychiatric symptoms occurring after a brain lesion (Fox 2018). Rather than focusing on lesion locations alone, this approach explores the network of regions functionally connected to each lesion, using resting state functional connectivity from large databases of healthy individuals. Since it was first published, LNM has been a valuable tool to increase and improve knowledge, at the functional network level, about complex post-lesional neuropsychiatric syndromes (Fox 2018). A recent example of the application of LNM is lesional mania, a heterogenous neuropsychiatric syndrome characterized by expansive mood, increased energy, grandiosity, impaired thinking, and poor judgment (Cotovio et al. 2020). In that study, we showed that, while lesions associated with mania are distributed across several brain areas (Barahona-Corrêa et al. 2020), these lesion locations have a unique pattern of functional connectivity to the right orbitofrontal cortex, right inferior temporal gyrus and right frontal pole, a connectivity profile that was robust across different validation strategies and aligned with brain stimulation targets associated with induction or alleviation of mania symptoms.

Nevertheless, the validity and reproducibility of LNM have been challenged (e.g., Cohen et al. 2019; Bobes et al. 2021) and one of the most important methodologic constraints that has been consistently pointed out is the potential impact of performing lesion network mapping using different sources of resting state functional magnetic resonance imaging (rs-fMRI) data. In fact, different connectomes may vary in size, field of strength and acquisition time, among other characteristics (Cohen et al. 2021; Cohen and Fox 2021). Such differences were hypothesized to potentially impact the results of neuroimaging analyses, including lesion network mapping, hence decreasing their validity and reliability (Cohen et al. 2021). Given the importance of this question, and the fact that it is empirically tractable, we became interested in understanding if our previously published LNM results for lesional mania are reproducible when using different connectomes. To address this question, we returned to our original lesional mania map, as reported in Cotovio et al. (2020), for comparison with validation lesional mania maps, obtained using different methods, including different connectomes.

## Methods

To obtain the validation lesional mania maps, we performed lesion network mapping as described in Cotovio et al. (2020), but using normative rs-fMRI obtained from several different connectomes. Specifically, we used data from: 937 subjects from the Human Connectome Project (HCP; http://www.humanconnectome.org/study/hcp-young-adult/) (Glasser et al. 2013), scanned on a 3 Tesla MRI scanner (“HCP 3T 937”); a smaller size connectome of 155 randomly selected subjects of the original HCP (“HCP 3T 155”), matching the smallest connectome we had available (please see below); 937 subjects from the HCP but shortening the original time series to 124 time points i.e., 1 min 29 s, (“HCP 3T 937 shortened”); 155 subjects from the HCP, scanned on a 7 Tesla MRI scanner; 189 subjects from a different connectome, the Max Planck Institute (MPI)-Leipzig Mind Brain Body (MBB; https://ftp.gwdg.de/pub/misc/MPI-Leipzig_Mind-Brain-Body/) (Mendes et al. 2019), scanned on a 3 Tesla MRI scanner, but which have used a different preprocessing pipeline that did not include global signal regression (“MBB”), similar to the connectome used by Bobes and colleagues (Bobes et al. 2021). As before (Cotovio et al. 2020), the network map of lesional mania locations was statistically compared to that derived from locations of control lesions, not associated with mania, to compute a final map identifying connections differing significantly between mania and control lesions. Again, control lesion locations consisted of both two-dimensional (2D) literature-derived images, as well as three-dimensional (3D) MRI images from clinical cohorts. Here, while 2D controls were the same as those used in Cotovio et al. (2020), 3D controls were replaced by images from a more heterogenous cohort, comprising 608 lesions not selected for any specific symptom, namely brain tumors and stroke lesions, from the BRATS dataset (Menze et al. 2014) and the ATLAS R 1.2 dataset (Liew 2018), respectively. The final lesional mania cohort included 56 patients (2D: *N*=41; 3D: *N*=15), while the control lesion cohort had 687 patients (2D: *N*=79; 3D: *N*=608). Median lesion volume in the lesional mania cohort was 313 voxels (interquartile range [IQR]: 93–875) for 2D lesions and 5725 voxels (IQR: 1486–10,189) for 3D lesions, while in control lesions sample, it was 32 voxels (IQR: 14–80) for 2D lesions and 14,562 voxels (IQR: 1816–29,369) for 3D lesions. As detailed in Cotovio et al. (2020), for each lesion, we computed correlations of the rs-fMRI time-course between the average activity for the lesion location and the activity of every voxel in the brain, using data from each MRI in the aforementioned connectomes. To obtain the network map for each lesion, we then averaged the results across all MRIs from each connectome. Finally, to identify differences in connectivity between mania and control lesions, Fischer z transformed correlation maps for the mania lesions (i.e., network maps) were compared with those for control lesions. The statistical comparison between mania and control network maps was performed using a voxel-wise permutation-based two-sample t test implemented within FSL PALM, with five thousand permutations per test, threshold-free cluster enhancement (TFCE), two-tailed testing, and *α*<0.05 with family-wise error (FWE) correction for multiple comparisons. Importantly, as ratio and lesion size of 2D and 3D lesions of the two cohorts were significantly different, all maps were computed controlling for lesion size and dimensionality, to guarantee that differences in lesion size or dimensionality were not driving the connectivity differences between mania and control lesions. The connectivity patterns in the maps resulting from case vs. control comparisons are viewed as having increased specificity for functional connectivity of cases (Cotovio et al. 2020; Ferguson et al. 2019). Specifically, the mania vs. control maps will reveal regions that are significantly more connected to mania lesions than control lesions, shown in warm colors, as well as regions that are significantly more connected to control lesions than mania lesions, which are represented in cool colors. Finally, to test similarity between the maps resulting from the original connectome and those from the different connectomes, we calculated spatial correlation between the uncorrected maps using Pearson’s correlation, since data was normally distributed. In an exploratory analysis, FWE corrected maps were also used. Statistical significance for spatial correlation was defined using non-parametric permutation testing approach, as supported by others (Siddiqi et al. 2021; Castro-Rodrigues et al. 2020). Spatial correlation was re-computed 10,000 times after randomizing data across the connectivity maps, obtaining the null distribution. *P* values were defined as the proportion of randomly permuted results that were more extreme than the real result (Siddiqi et al. 2020).

## Results

**Fig. 1:**
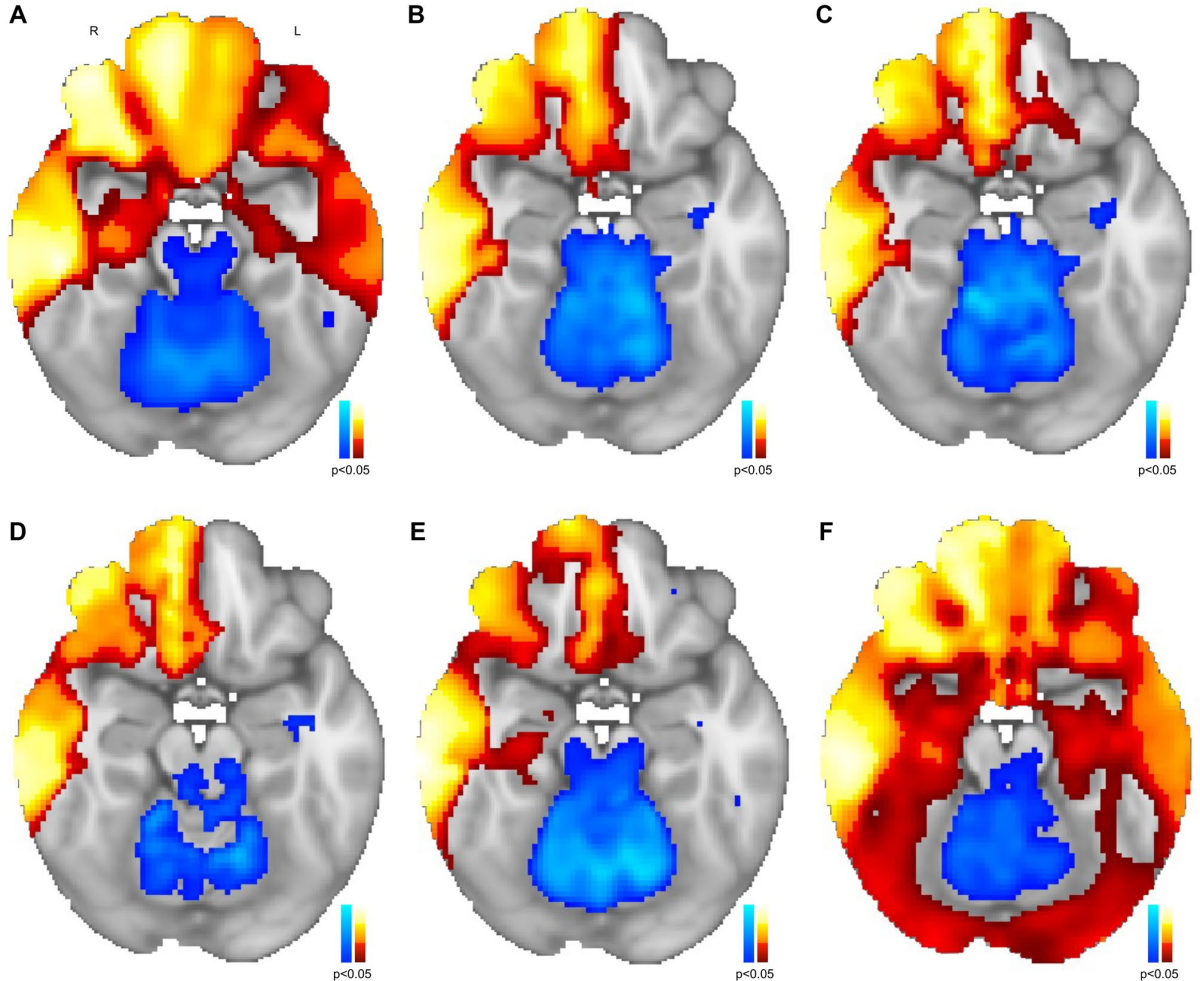
**Lesional network mapping produced similar results across different connectomes.** When compared to the resulting map from the original connectome (**A**), the validation lesional network maps produced similar results when using different connectomes: HCP 3T 937 (**B**); HCP 3T 155 (**C**); HCP 3T 937 shortened (**D**); HCP 7T (**E**); MBB (**F**). A strong spatial correlation, Pearson’s r varying from 0.85 to 0.99, between all lesional mania maps was obtained when comparing all network maps

**Fig. 2:**
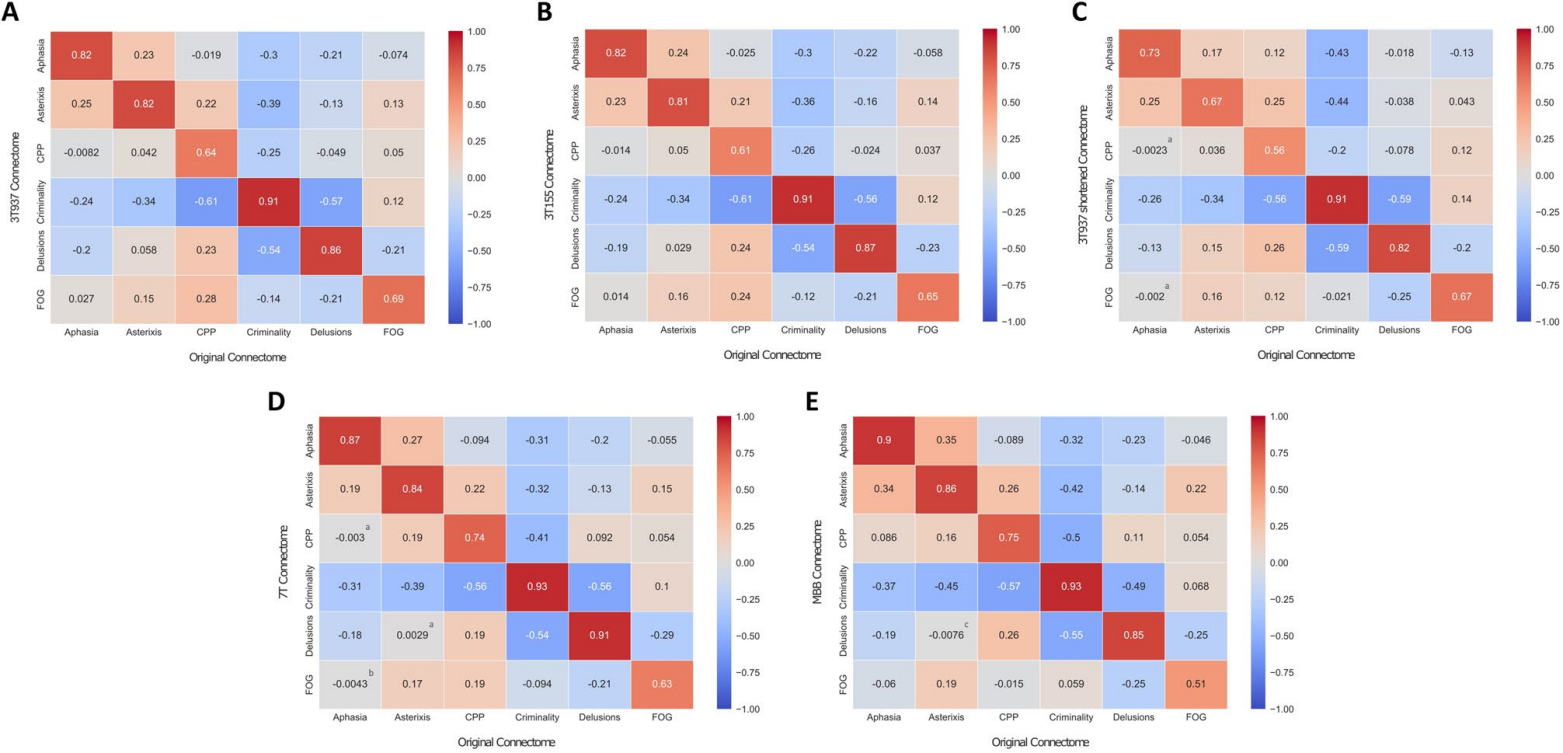
**Lesional network mapping produced similar results across different neuropsychiatric syndromes when using different connectomes. **Across several different neuropsychiatric syndromes, similar findings were observed when comparing the lesional network maps resulting from the original connectome to those produced using different connectomes: HCP 3T 937 (**A**); HCP 3T 155 (**B**); HCP 3T 937 shortened (**C**); HCP 7T (**D**); MBB (**E**). Each panel represents the spatial correlation coefficients (*r*) of the pairwise comparisons between distinct lesional connectivity maps. All correlations values were significant at *p*<0.0001, unless noted otherwise (^a^non-significant; ^b^*p*<0.05; ^c^*p*<0.001). *3T* Three Tesla; *7T* Seven Tesla; *CPP* Central Post-stroke Pain; *FOG* Freezing of Gait; *HCP* Human Connectome Project; *MBB* Mind Brain Body

**Fig. 3:**
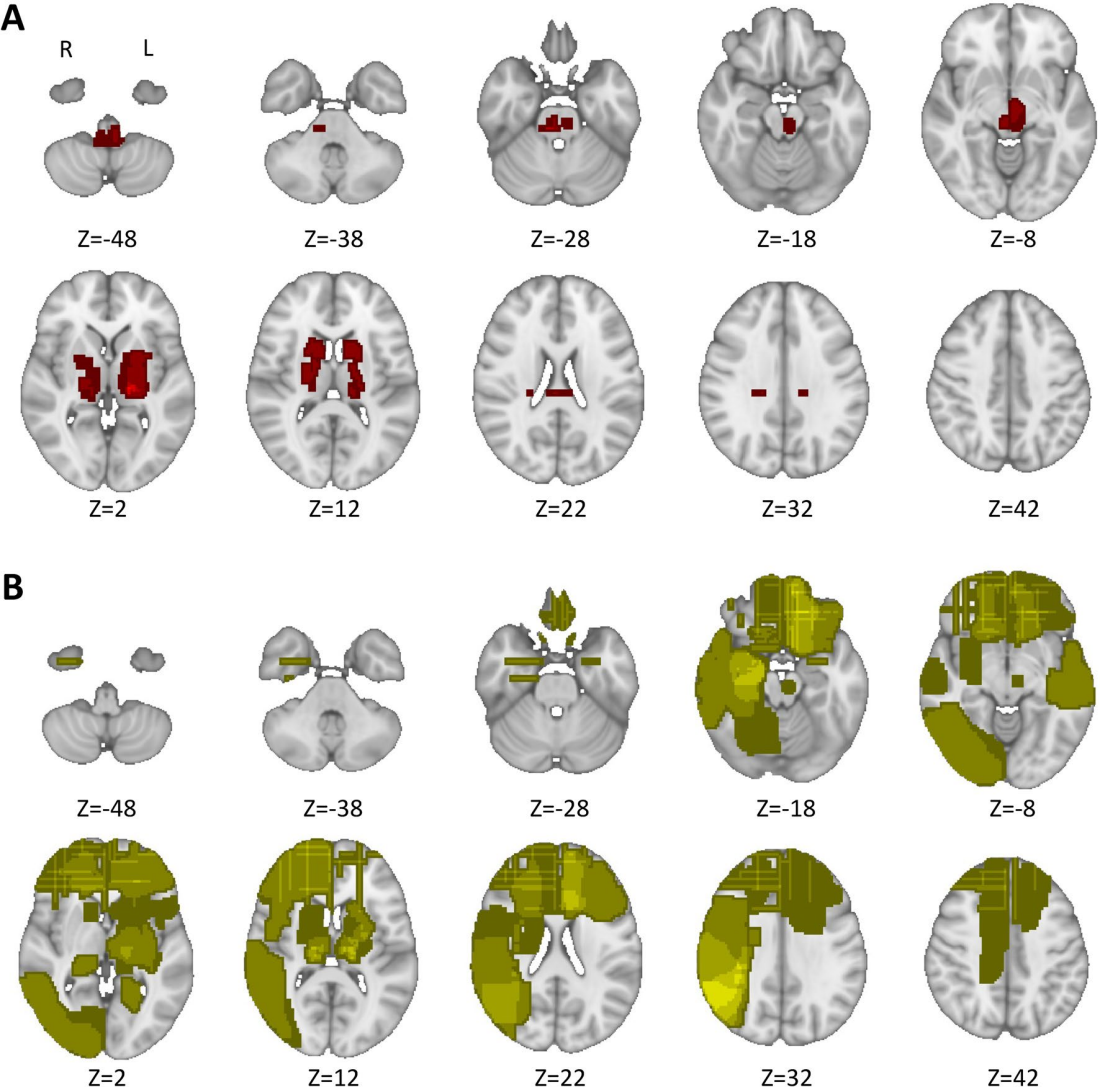
**Lesion topography may moderate lesional network mapping reproducibility.** In neuropsychiatric syndromes with moderate reproducibility across connectomes (spatial correlation=0.5–0.7), lesions were preferably located in deeper brain regions (**A**). Conversely, in those with stronger agreement (spatial correlation>0.7), lesions were more frequently located across different cortical regions (**B**). On both lesion overlap maps we have applied 1 mm Full-Width at Half Maximum spatial smoothing, using Python 3 *nilearn,* for visualization purposes

Despite obtaining the validation lesional mania maps using different connectomes as well as a distinct and more heterogenous control sample, the maps were very similar to the original lesional mania map (Fig. 1). In fact, we found that spatial correlations across all lesional mania maps were very high (Pearson’s r varied from 0.85 to 0.99), reflecting a very strong agreement between all lesional mania maps. The spatial correlation between the original map and each new connectome map were the following: HCP 3T 937–0.87; HCP 3T 155–0.86; HCP 3T 937 shortened – 0.88; HCP 7T – 0.90; MBB – 0.89. All correlation values were significant (*p*<0.0001) and higher than 0.7, above which correlations are considered to be strong/very strong (Akoglu 2018). We then re-computed the spatial correlations between the original map and each new connectome map but using the *p*<0.05 FWE corrected maps instead. While overall spatial correlation values decreased (HCP 3T 937–0.75; HCP 3T 155–0.75; HCP 3 T 937 shortened – 0.74; HCP 7T – 0.80; MBB – 0.81), they were still significant (*p*<0.0001) and higher than 0.7. Finally, to test the specificity of the current LNM methods, we have applied the same case vs. controls LNM analysis strategy to other symptoms/syndromes (aphasia, *N*=12; asterixis, *N*=30, central poststroke pain, *N*=23; criminal behavior, *N*=17; delusions, *N*=15; freezing of gait, *N*=14) according to cases included in previous publications (Boes et al. 2015; Laganiere et al. 2016; Darby et al. 2018, 2017). In each case, the remaining syndromes and 2D mania were used as controls. We compared the resulting maps using cross-correlograms between maps resulting from the original connectome and those from other connectomes. We found moderate-to-very strong agreement when comparing maps for the same syndromes produced using different connectomes, but poor reliability when different syndromes were compared (Fig. 2). Interestingly, considering same syndrome comparisons, in those syndromes revealing moderate reproducibility across connectomes (spatial correlation=0.5–0.7), lesions were preferably located on deeper brain regions, while in those with higher agreement (spatial correlation>0.7), lesions were more frequently located across cortical regions (Fig. 3).

## Discussion

Our results highlight that, given a set of lesions associated with a specific neuropsychiatric syndrome, current methods for LNM are reliable and robust to the impact of critical methodologic choices, namely the connectome and the control lesions, with highly overlapping outcomes, even when both parameters are changed, or when including lesions with different characteristics such as lesion size and dimensionality. In fact, when considering the latter, previous work has consistently shown that lesion networks derived from 2D slices are very similar to networks obtained from 3D lesions (Cotovio et al. 2020; Boes et al. 2015; Sutterer et al. 2016). Interestingly, high reproducibility was also found even when using a connectome without global signal regression (MBB, Fig. 1F), a preprocessing step that has been a matter of debate (Murphy and Fox 2017). Despite the overall high spatial correlation with the original map when global signal regression was not performed, wider positive regions were found, which may further support the use of global signal regression to improve the specificity of positive correlations (Murphy and Fox 2017; Fox et al. 2009). Hence, global signal regression may increase the likelihood of finding specific regions in the brain that when lesioned will be more (network warm colored nodes) or less (network cool colored nodes) associated to the occurrence of a specific neuropsychiatric syndrome (Murphy and Fox 2017; Fox et al. 2009; Cotovio et al. 2020; Ferguson et al. 2019). There is heterogeneity in thresholds used for LNM across different studies (Cohen and Fox 2021; Sperber and Dadashi 2020), potentially affecting the results of the analyses performed. Nevertheless, we found that, for current LNM methods, i.e., comparing cases and controls network maps using voxel-wise permutation-based two-sample t tests, when using more stringent final mania vs. control maps, while overall reliability decreased, strong/very strong agreement was still found. Finally, we also found that our findings were consistent and reliable across different neuropsychiatric symptoms/syndromes. Interestingly, while still moderately reproducible, spatial agreement decreased in syndromes where lesions are preferably located on deeper brain regions. Such finding might be related to poorer fMRI signal reliability across different scanners observed in deeper brain structures (Zhao et al. 2018). Nevertheless, this and other hypotheses should be further explored in future studies using LNM.

All these features are of critical importance for the use of LNM to study the pathophysiology of lesional neuropsychiatric syndromes (Boes et al. 2015; Laganiere et al. 2016), such as Capgras Syndrome (Darby et al. 2017), amnesia (Ferguson et al. 2019) or prosopagnosia (Cohen et al. 2019), as well as to clarify the neurobiological substrate for the therapeutic effects of different neuromodulation strategies (Siddiqi et al. 2021), among other objectives (Fox 2018). Moreover, it is also a fundamental property of LNM while it evolves as a potential clinical predictive tool, i.e., as a potential approach that can estimate the impact of brain lesions in impairing the functions of different regions and networks. We believe that these results contribute to the important discussions about past, present, and future of LNM (Boes 2021).

Nevertheless, LNM is not without limitations, which have been recently pointed out and discussed by several authors, mainly concerning its validity as a predictive tool for behavioral outcomes after brain lesions (Salvalaggio et al. 2020, 2021; Umarova and Thomalla 2020), as well as the impact of different study designs and methodologies, such as sample size or statistical threshold, on network analysis outcomes (Sperber and Dadashi 2020). The different authors highlight that current LNM practice offers several methodologic challenges that should be addressed (Poldrack et al. 2020) to improve and validate LNM as a clinical tool to predict outcomes resulting from brain lesions. We believe that the evidence of high reproducibility across the different potential methodologic constraints tested here, including the use of different connectomes, is one of such important steps for considering future prospective research regarding clinical use of LNM.

## Data Availability

Data availability is not applicable to this article as no new data were created or analyzed.
